# Genomic rearrangements in *BRCA1* and *BRCA2*: A literature review

**DOI:** 10.1590/S1415-47572009005000049

**Published:** 2009-09-01

**Authors:** Ingrid Petroni Ewald, Patricia Lisboa Izetti Ribeiro, Edenir Inêz Palmero, Silvia Liliana Cossio, Roberto Giugliani, Patricia Ashton-Prolla

**Affiliations:** Laboratório de Medicina Genômica, Hospital de Clínicas de Porto Alegre, Porto Alegre, RSBrazil; 2 Programa de Pós-Graduação em Medicina: Ciências Médicas, Universidade Federal do Rio Grande do Sul, Porto Alegre, RSBrazil; 3 Faculdade de Medicina, Universidade Federal do Rio Grande do Sul, Porto Alegre, RSBrazil; 4 Programa de Pós-Graduação em Genética e Biologia Molecular, Universidade Federal do Rio Grande do Sul, Porto Alegre, RSBrazil; 5 Programa de Pós-Graduação em Medicina: Ciências Gastroenterológicas, Universidade Federal do Rio Grande do Sul, Porto Alegre, RSBrazil; 6 Instituto Nacional de Genética Médica Populacional, Porto Alegre, RSBrazil; 7 Serviço de Genética Médica, Hospital de Clínicas de Porto Alegre, Porto Alegre, RSBrazil

**Keywords:** *BRCA1*, *BRCA2*, breast cancer, genomic rearrangements, MLPA

## Abstract

Women with mutations in the breast cancer genes *BRCA1* or *BRCA2* have an increased lifetime risk of developing breast, ovarian and other *BRCA*-associated cancers. However, the number of detected germline mutations in families with hereditary breast and ovarian cancer (HBOC) syndrome is lower than expected based upon genetic linkage data. Undetected deleterious mutations in the *BRCA* genes in some high-risk families are due to the presence of intragenic rearrangements such as deletions, duplications or insertions that span whole exons. This article reviews the molecular aspects of *BRCA1* and *BRCA2* rearrangements and their frequency among different populations. An overview of the techniques used to screen for large rearrangements in *BRCA1* and *BRCA2* is also presented. The detection of rearrangements in *BRCA* genes, especially *BRCA1,* offers a promising outlook for mutation screening in clinical practice, particularly in HBOC families that test negative for a germline mutation assessed by traditional methods.

## Introduction

The precise identification of germline *BRCA1* and *BRCA2* mutations is a major concern for geneticists counseling families with a high risk of breast and ovarian cancers. The most frequent mutations encountered in these genes are deletions or insertions of a few bases or single-base substitutions that result in premature stop codons (Perrin-Vidoz *et al.*, 2002; Narod and Foulkes, 2004). Such point mutations occur throughout the coding sequence of both genes and account for 10%-50% of the germline mutations encountered in hereditary breast and ovarian cancer (HBOC) families, depending on the inclusion criteria used (Agata *et al.*, 2005; Vasickova *et al.*, 2007).

The observed frequencies of *BRCA1* mutations are lower than predicted by linkage analysis, with pathogenic variations in the coding region or splice sites of the gene being found in approximately two-thirds of *BRCA1*-linked families. This finding suggests that other dominant genes (Ford *et al.*, 1998; Armour *et al.*, 2002) and/or low penetrance alleles, such as the 1100delC mutation in *CHEK2*, may be associated with the HBOC phenotype (Puget *et al.*, 1999; Nevanlinna and Barker, 2006). Indeed, breast and ovarian cancers have been associated with germline mutations in other genes that are involved in the maintenance of genomic integrity, such as *TP53, PTEN, ATM, NBS1, RAD50, BRIP1* and *PALB2.* Inherited breast cancer is currently considered a highly heterogeneous genetic disease with respect to both the *loci* and alleles involved (Walsh *et al.*, 2006; Walsh and King, 2007).

Large genomic rearrangements have recently been identified in HBOC families and account for a small but still significant proportion of cases in several populations. These mutations are usually pathogenic because deletions or insertions of large genomic sequences within a coding region result in out-of-frame translation and usually lead to a mutant peptide of abnormal structure and/or function (Preisler-Adams *et al.*, 2006). These mutations may be overlooked by most of the available screening and diagnostic PCR-based methods that use qualitative rather than quantitative methods and do not detect partial or complete exon losses or gains (Armour *et al.*, 2002). Large genomic rearrangements of *BRCA1* may account for up to one-third of all disease-causing mutations in various populations, while large genomic rearrangements in *BRCA2* are less frequently observed (Hansen *et al.*, 2009).

## Frequency of Large Rearrangements

As shown in Table 1, the frequency of large genomic rearrangements varies considerably among populations. Among HBOC families, the highest proportion of *BRCA1* rearrangements has been observed in northern Italy, where large genomic deletions account for approximately one-third of the pathogenic *BRCA1* mutations (Montagna *et al.*, 2003) and the overall prevalence of rearrangements in the families studied is 23%. In the Netherlands, rearrangements also represent a high proportion of all deleterious mutations in *BRCA1* (27%-36% of all germline mutations in the gene) and are attributable to founder mutations (Petrij-Bosch *et al.*, 1997; Hogervorst *et al.*, 2003). In contrast, western Danish families with HBOC have a *BRCA1* rearrangement prevalence of 3.8% (Thomassen *et al.*, 2006). Another study done in Finland failed to detect any rearrangements among 82 families with moderate or high risk for HBOC (Lahti-Domenici *et al.*, 2001). The latter two studies indicate a lower frequency of genomic rearrangements in Nordic countries. Finally, a study in Canada found no evidence of *BRCA1* or *BRCA2* genomic rearrangements in high-risk French-Canadian breast/ovarian cancer families (Moisan *et al.*, 2006).

This wide range in the prevalence of rearrangements is most likely related to the different genetic backgrounds of the populations studied, although the heterogeneity of the clinical inclusion criteria used for HBOC in each study may also have influenced the results. Furthermore, the prevalence of rearrangements will be different in samples that include only *BRCA* mutation-negative individuals by sequencing compared to those that include previously untested individuals at risk for HBOC. More recent studies have encountered an intragenic rearrangement prevalence of 6% and 12%, respectively, in high-risk patients in families from the Czech Republic and the United States of America who were negative for *BRCA1/2* point mutations by sequencing (Walsh *et al.*, 2006; Vasickova *et al.*, 2007). In Germany, the prevalence of *BRCA1* rearrangements is lower, ranging from 1 in 59 (1.7%) to 1 in 17.5 (5.7%) among high-risk families who are mutation-negative by sequencing (Hofmann *et al.*, 2003; Hartmann *et al.*, 2004; Preisler-Adams *et al.*, 2006).

Only a few studies have examined the prevalence of *BRCA2* rearrangements in larger sets of high-risk patients. In a report from Australia, large genomic rearrangements in *BRCA2* were identified in 2% of 149 high-risk families that tested negative for *BRCA1* and *BRCA2* point mutations (Woodward *et al.*, 2005). Agata *et al.* (2005) found a similar frequency (2.5%) of *BRCA2* rearrangements among 121 highly selected Italian families. In a recent study of Portuguese HBOC families, a single founder *BRCA2* rearrangement (c.156_157insAlu) was identified in 8% of the families studied and is the most frequent *BRCA2* rearrangement described to date (Machado *et al.*, 2007).

## Molecular Pathology of *BRCA1* Rearrangements

Several *BRCA1* germline rearrangements with well characterized breakpoints have been reported (Mazoyer, 2005). These rearrangements are scattered throughout the gene and although most of them are deletions, duplications, triplications or combined deletion/insertion events have also been described. The *BRCA1* gene characteristically has an extremely high density of intronic *Alu* repeats and a duplicated promoter region containing a *BRCA1* pseudogene that most likely account for the occurrence of “*hot spots*” that favor unequal homologous recombination events (Smith *et al.*, 1996; Puget *et al.*, 2002). Currently, 45 different large genomic rearrangements have been characterized worldwide, including deletions and duplications of one or more exons (Table 1).

### *Alu* sequences

**Figure 1:**
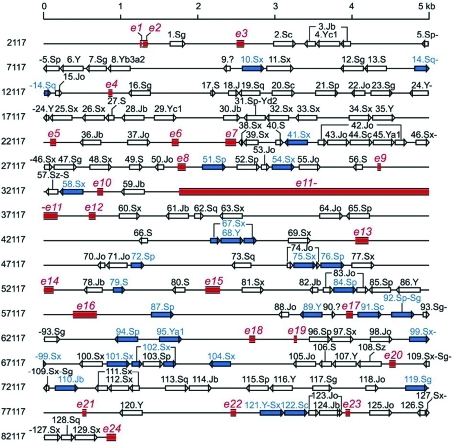
*Alu* elements in *BRCA1* (reproduced from Pavlicek *et al.* 2004, by permission of Oxford University Press). Exons are depicted as red rectangles and *Alu* sequences as arrows. *Alu* elements known to be involved in human exonic deletions and/or duplications are shown in blue.

The human genome contains up to 1 million copies of interspersed *Alu* elements (approximately one *Alu* repeat for every 5 kb) that apparently mediate chromosomal rearrangements and homologous recombination events, resulting in translocations, duplications, inversions or deletions (Kolomietz *et al.*, 2002; Tancredi *et al.*, 2004). These sequences are named *Alu* because most of the members of this family of repeats are cleaved by the bacterial restriction endonuclease *AluI.* Members of the *Alu* family show significant homology but do not have identical sequences. Around 500,000 members of the *Alu* family have been identified and it is estimated that together they comprise 3% of the human genome. Approximately 41.5% of the intronic sequences of *BRCA1* consist of *Alu* elements (Figure 1) that range in size from 0.5 kb to 23.8 kb and are located throughout the entire gene (Montagna *et al.*, 1999).

*Alu* sequences have often been regarded as genomic instability factors because they are responsible for recombinational “*hot spots*” in certain genes and are frequently involved in exon shuffling during meiosis as a result of non-homologous recombination. These sequences may also act as regulatory factors in transcription, with structural roles (as “physical separators" of protein-protein interactions during chromosome condensation in cellular division) and functional roles (in alternative “splicing” or as a connection between transcription factors) being proposed.

The two most prevalent sub-classes of repetitive elements in the *Alu* family are LINEs (Long Interspersed Elements) and SINEs (Short Interspersed Elements). LINEs span 6-8 kb and represent ~21% of the total human genome DNA, whereas SINEs, which are derived from RNA polymerase transcripts, are shorter (100-300 bp) and represent ~13% of the human genome. LINEs and SINEs are mobile elements that move via reverse transcription (Gad *et al.*, 2001).

The complete genomic sequence of *BRCA1* was published by Smith *et al.* (1996), who identified 138 individual *Alu* elements within this gene. Rearrangements are less common in the *BRCA2* gene, probably because of a lower frequency of *Alu* sequences (17%). In most of the well characterized rearrangements described in the literature, there is good evidence for the involvement of *Alu* repeat elements in the recombination event. For example, the *BRCA1* exon 5-7 deletion described in German families results from a non-allelic homologous recombination between *AluSx* in intron 3 and *AluSc* in intron 7. Both *Alu* repeats share a homologous region of 15 bp at the crossover site. (Preisler-Adams *et al.*, 2006)

###  Non-functional pseudogenes

Another important cause of unequal recombination within the coding region of certain genes is the presence of non-functional pseudogenes with high sequence homology to at least parts of the functional gene. Pseudogenes are usually non-functional “relatives” of known genes that have lost their protein-coding ability or are no longer expressed in the cell (Vanin, 1985).

Puget *et al.* (2002) were the first to report this mutational mechanism for the *BRCA1* gene. In two families with HBOC, these authors showed that the first exons of the gene were replaced by those of the *BRCA1* pseudogene, ψ*BRCA1*. This pseudogene had previously been shown to lie ~30 kb upstream of *BRCA1* (Barker *et al.*, 1996; Brown *et al.*, 1996). The presence of a duplication containing most of *BRCA1* exons 1 and 2 and the identification of two different recombination events involving homologous regions located in the *BRCA1* gene and ψ*BRCA1*, respectively, led the authors to postulate that these regions were strong “*hot spots*” for recombination. The mutant alleles identified in the study harbored a chimeric gene that consisted of ψ*BRCA1* exons 1A, 1B, and 2 fused to *BRCA1* exons 3-24. This chimeric gene lacked both the *BRCA1* promoter and translation initiation codon and was therefore non-functional (Hofmann *et al.*, 2003).

###  Tandemly arranged short sequence repeats

Gross chromosomal deletions and/or insertions may also be mediated by tandemly arranged short sequence repeats. Highly repetitive nonconding human DNA often occurs in arrays (or blocks) of tandem repeats of sequences which may be simple (1-10 nucleotides) or moderately complex (tens to hundreds of nucleotides). Individual arrays can occur at a few or many different chromosomal locations. Satellite DNA, which constitutes most of the heterochromatic regions of the genome and is particularly noticeable in the vicinity of centromeres, consists of very large arrays of tandemly repeated DNA. Short repeats may cause slipped mispairing during replication, resulting in deletions or duplications of varying sizes. Recombination involving tandemly arranged short sequence repeats underlies the 244 bp deletion in *BRCA1* exon 5 described in German HBOC families (Preisler-Adams *et al.*, 2006).

## *BRCA2* Rearrangements

Only a few studies have investigated the presence and frequencies of deleterious *BRCA2* rearrangements, and most of these were either done on a relatively small number of families or used cumbersome mutation detection methods of variable sensitivity (Agata *et al.*, 2005).

Until recently, only two genomic rearrangements had been identified in six studies that analyzed hereditary breast cancer patients or primary breast tumors among diverse European populations (Peelen *et al.*, 2000; Lahti-Domenici *et al.*, 2001; Chin *et al.*, 2001; Wang *et al.*, 2001; Gad *et al.*, 2002; Bunyan *et al.*, 2004). The greatly reduced incidence of large genomic alterations that affect *BRCA2* compared to *BRCA1* most likely reflects differences in the density of *Alu* repeat sequences at the two *loci,* and these initial studies were not very supportive of the inclusion of this type of analysis in routine mutation testing of HBOC families (Preisler-Adams *et al.*, 2006).

To date, 16 *BRCA2* germline rearrangements have been reported. More recent studies have reported the frequent occurrence of large genomic *BRCA2* rearrangements in male breast cancer families. Woodward *et al.* (2005) reported three *BRCA2* rearrangements in 25 families with at least one male breast cancer, but no *BRCA2* rearrangements in 114 families without male breast cancer, and Tournier *et al.* (2004) described three *BRCA2* rearrangements in 39 French families with at least one case of male cancer. These findings indicate that large genomic rearrangements in *BRCA2* are more frequent in families with male breast cancer.

Another recent study done in Portugal described a common *BRCA2* rearrangement involving an *Alu* element, c.156_157ins*Alu* in exon 3, in 17 (8%) of 210 HBOC families (Machado *et al.*, 2007).

## Methods for Detecting Rearrangements

Classic methods for mutation detection (such as sequencing) are usually unable to identify large genomic rearrangements. Consequently, several alternative methods have been developed for the analysis of structural genomic abnormalities. These methods, which are designed to target either one or a few specific *loci*, or to scan the whole genome, include Southern blotting, long-range PCR, fluorescent *in situ* hybridization (FISH), quantitative multiplex PCR of short fluorescent fragments (QMPSF), protein truncation test (PTT), comparative genomic hybridization (CGH), real-time or quantitative PCR (RT-PCR or qPCR) and multiplex ligation-dependent probe amplification (MLPA). Although each of these methods has potential advantages and limitations, there have been very few large-scale comparative analyses of these techniques. A brief summary of the most common detection methods is provided below.

###  Southern blotting

Southern blotting is the transfer of DNA fragments from an electrophoretic gel to a membrane support that results in immobilization of the fragments on the membrane and in a semipermanent reproduction of the banding pattern of the gel. This technique can be used to detect changes in copy number (deletions and duplications) when samples are run in parallel (concomitantly) with an internal standard. In addition, large rearrangements may also be detected by a size shift in the blotted DNA fragments. Although frequently used in the past, this method has lost popularity as a routine diagnostic procedure since it is laborious, time consuming, requires large amounts of high-molecular weight DNA and its interpretation may be hampered by false-negative results (Unger *et al.*, 2000; Brown, 2001; De Lellis *et al.*, 2007).

###  Long-range PCR

Long-range PCR uses a mixture of two thermostable DNA polymerases (proofreading and non-proofreading), thereby increasing the product size to 35 kb. The method has been useful for identifying specific large aberrations, including intragenic deletions, insertions, duplications and chromosomal breakpoints in several disorders. Long-range PCR was originally designed to detect changes in gene copy number rather than translocations or inversions, requires small amounts of DNA and is excellent for locus-specific identification of known rearrangements. These features make it ideal for diagnostic purposes. However, this technique is limited by its low throughput and is unable to provide a genome-wide view of rearrangements, which therefore restricts its usefulness to the analysis of a specific genomic region delimited by the primers that are used (Vasickova *et al.*, 2007; Morozova and Marra, 2008).

###  Fluorescent *in situ* hybridization (FISH)

FISH is based on the hybridization of fluorescent probes to metaphase or interphase nuclei followed by analysis with a fluorescence microscope. FISH can detect variations in copy number (deletions and duplications), translocations and inversions. Copy number is assessed by microscopic visualization. The most commonly used conventional *in situ* hybridization protocol in cancer research is dual-color FISH. This method involves labeling centromeres and the DNA region of interest with different colors and estimating the probe copy number from the ratio of the centromeric to noncentromeric signal. Dual-color FISH is used to detect chromosomal gains or losses (aneuploidy), intrachromosomal insertions, deletions, inversions, amplifications and chromosomal translocations. The advantages of FISH include the ability to analyze single cells, applicability to a wide range of substrates, including fixed samples (such as paraffin-embedded tissue), and relative simplicity of use. The method cannot provide a genome-wide assessment of DNA rearrangements, with the exception of gross chromosomal aberrations detected by multifluor-based techniques, and is thus of limited value for genome-wide identification of smaller-scale chromosomal aberrations (De Lellis *et al.*, 2007; Morozova and Marra, 2008).

###  Quantitative multiplex PCR of short fluorescent fragments (QMPSF)

QMPSF is a sensitive method for the detection of genomic deletions or duplications based on the simultaneous amplification of short genomic fragments using dye-labelled primers under quantitative conditions. The PCR products are analyzed on a sequencing platform used in the fragment analysis mode and the peak height and area are proportional to the quantity of template present for each target sequence. In this setting, the height or area of peaks corresponding to the loss of one allele will be half that of normal samples, whereas a gain of one allele will result in a 50% increase. This method is rapid and sensitive and has been used to screen for *BRCA1* rearrangements (Casilli *et al.*, 2002; Bastard *et al.*, 2007; Weitzel *et al.*, 2007). However, it is not easily implemented in a routine mutation analysis laboratory and requires a fair amount of previous experience.

###  Protein truncation test (PTT)

The PTT method is a straightforward approach to screen for biologically relevant gene mutations. The method is based on the size analysis of products resulting from transcription and translation *in vitro*. Proteins of lower mass than the expected full-length protein represent translation products derived from truncating frameshift or nonsense mutations in the analyzed gene. Mutation detection may be limited by the size and location of the rearrangement in relation to the primers used in the assay. In addition, because of the low sensitivity of conventional PTT, mutations can be detected only in samples that harbor a relatively high number of mutated gene copies (Peelen *et al.*, 2000; Hauss and Müller, 2007).

###  Comparative genomic hybridization (CGH)

CGH (also known as chromosomal microarray analysis or CMA) is a molecular-cytogenetic method that has been used to analyze variations in copy number (gains or losses) of DNA from patients and/or tumor cells. The method is based on the hybridization of fluorescently labeled tumor DNA and normal DNA to normal human metaphase preparations. Using epifluorescence microscopy and quantitative image analysis, regional differences in the fluorescence ratio of gains/losses *vs.* control DNA can be detected and used to identify abnormal regions in the genome. CGH does not identify structural chromosomal aberrations such as balanced reciprocal translocations or inversions since they do not change the copy number. Although CGH is a complex technique that requires significant previous experience in cytogenetics and a specific set-up in terms of infra-structure, it is an efficient method for genome-wide screening of rearrangements (Rouleau *et al.*, 2007).

###  Real time polymerase chain reaction (qPCR)

Real time PCR, also known as quantitative real time polymerase chain reaction (qPCR), is a polymerase chain reaction-based technique used to amplify and simultaneously quantify a target DNA molecule. qPCR allows the detection and quantification (as absolute number of copies or relative amount when normalized to DNA input or additional normalizing genes) of a specific sequence in a DNA sample. The procedure follows the general principle of PCR, the key difference being that the amplified DNA is quantified as it accumulates in the reaction in *real time* after each amplification cycle. Two common methods of quantification are the use of fluorescent dyes that intercalate with double-stranded DNA, and modified DNA oligonucleotide probes that fluoresce when hybridized with a complementary DNA. Although this method is rapid and does not require a large amount of starting material, it has a limited throughput. It is not suitable for the detection of translocations or inversions or for genome-wide screening of rearrangements (Barrois *et al.*, 2004; Morozova and Marra, 2008).

###  Multiplex ligation-dependent probe amplification (MLPA)

MLPA is a multiplex PCR method developed to detect abnormal copy numbers of different genomic DNA sequences. Each MLPA probe consists of two oligonucleotides that can be ligated to each other when hybridized to a target sequence. All ligated probes have identical sequences at their 5' and 3' ends, permitting simultaneous amplification in a PCR containing only one primer pair. One of the two oligonucleotides of each MLPA probe has a common sequence used for PCR amplification at the 5' end and a target-specific sequence at the 3' end. The 5' region of the second oligonucleotide of each probe is designed to hybridise to the target sequence immediately adjacent to the first oligonucleotide and its 3' region has a common sequence used for PCR amplification and a “stuffer” sequence with different a specific length. Each probe gives rise to an amplification product of unique size, due to the variation in the stuffer sequence length. Because only ligated probes will be exponentially amplified during the subsequent PCR reactions the number of probe ligation products is a measure for the number of target sequences in the sample. The amplification products of different sizes are separated using capillary electrophoresis (Schouten *et al.*, 2002). Nevertheless, MLPA has certain drawbacks, including false-negative scores when probes are designed outside the region of interest, *i.e.*, outside the region involved in the rearrangement. MLPA is primarily used as a screening tool to identify rearrangements, and the precise location of the deletion or duplication breakpoints in the usually very large intronic or affected flanking regions must be refined by sequencing (Staaf *et al.*, 2008). In addition, in rare cases, MLPA may give a false-positive result for a deletion due to occurrence of a point mutation within the sequence of MLPA probe hibridisation (Gomez *et al.*, 2009). However, compared to most other techniques, MLPA is an inexpensive, sensitive, relatively simple, and high-throughput method (Hogervorst *et al.*, 2003; Dunnen and White, 2006; Ratajska *et al.*, 2008). The use of MLPA has facilitated the screening of genomic rearrangements in *BRCA1* (Montagna *et al.*, 2003; Hartmann *et al.*, 2004) and *BRCA2* (Woodward e*t al.*, 2005).

## Conclusion

**Figure 2:**
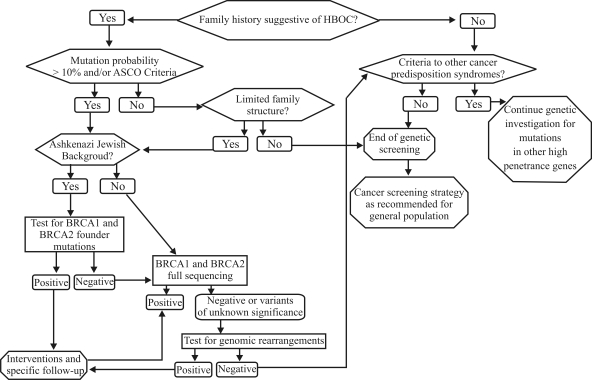
Suggested approach for molecular investigation of hereditary breast and ovarian cancer (HBOC) families. The mutation probabilities are estimated by using standard protocols and/or risk estimation tools such as BRCAPro, BOADICEA and the Myriad mutation prevalence tables. ASCO: American Society of Clinical Oncology.

Point mutations in the *BRCA* genes are the most common deleterious mutations encountered in HBOC families, and full gene sequencing and other PCR-based methods remain the gold standard for initial mutation identification. However, rearrangements in these genes have been described in a significant proportion of HBOC families, and are responsible for up to one-third of the identifiable *BRCA* mutations in certain populations. Consequently, in HBOC families that test negative for *BRCA* point mutations by conventional approaches, screening for large gene rearrangements in *BRCA1* and probably also *BRCA2* should be strongly considered. A suggested flowchart for investigation in these families is presented in Figure 2. The availability of relatively inexpensive and technically straightforward screening methods has greatly simplified this process, but often more than one method must be used to fully characterize a deletion or duplication in a given patient. Several studies in different populations have proven the usefulness of screening for *BRCA1* rearrangements, however the prevalence of such mutations in a given population should be known before definitive recommendations are made regarding the routine testing for rearrangements. In populations where there are highly prevalent founder rearrangements, preliminary screening for pathogenic *BRCA* gene mutations may be a cost-effective initial strategy.

## Figures and Tables

**Table 1:** Frequency of *BRCA1* and *BRCA2* genomic rearrangements among different populations.

Country	Gene studied	Prev *BRCA*	Prevalence	Proportion*	Rearrangements described	Reference
Australia	*BRCA1/2*	Yes	2%	-	*BRCA1*: Del. ex 3, ex 5, ex 21-23 *BRCA2*: Del. ex 1-2, ex 14-16	Woodward *et al.* (2005)
Canada	*BRCA1/2*	Yes	0%	0%	None	Moisan *et al.* (2006)
Czech Republic	*BRCA1*	Yes	6%	-	Del. ex 1A/1B-2, ex 5-14, ex 11-12, ex 18-19, ex 20, ex 21-22	Vasickova *et al.* (2007)
Denmark	*BRCA1/2*	Yes	1.3%	3.8%	*BRCA1:* Del. ex 3-16, ex 13-15	Thomassen *et al.* (2006)
Finland	*BRCA1/2*	Yes	0%	0%	None	Lahti-Domenici *et al.* (2001)
Germany	*BRCA1/2*	Yes Yes Yes	1.7-5.7%	8%	*BRCA1*: Del. ex 1A/1B-2, ex 5, ex 5-7, ex 17; Dupl. exon 13.	Hofmann *et al.* (2003), Hartmann *et al.* (2004), Preisler-Adams *et al.* (2006)
Italy	*BRCA1*	Yes	23%	40%	Del. ex 1A/1B-2, ex 9-19, ex 18-19, ex 20	Montagna *et al.* (2003)
Italy	*BRCA2*	Yes	2.5%	-	Del. ex 17-18, ex 8-11, ex 20	Agata *et al.* (2005)
Netherlands	*BRCA1*	Yes	7-9.1%	27%-36%	Del. ex 8, ex 13, ex 20-22, ex 22; Dupl. ex 13, ex 21-23; Tripl. ex 17-19	Petrij-Bosch *et al.* (1997), Hogervorst *et al.* (2003)
Poland	*BRCA1/2*	Yes	4.7%	4.5%	*BRCA1:* Del. ex 1A/1B-2, ex 17-19	Ratajska *et al.* (2008)
Portugal	*BRCA1*	Yes	9.6%	-	Del. ex 1-22, ex 8-13, ex 15-16; Dupl. ex 3-8, ex 18-20	Casilli *et al.* (2002)
Portugal	*BRCA1/2*	Yes	1.1%	6.7%	*BRCA1:* Del. ex 11-15	Peixoto *et al.* (2006)
Portugal	*BRCA2*	No	8%	-	Dupl. exon 3	Machado *et al.* (2007)
Portugal	*BRCA1/2*	Yes	1.1%	6.7%	*BRCA1:* Del. ex 11-15	Peixoto *et al.* (2006)
Singapore	*BRCA1/2*	Yes	3%	14.3%	*BRCA1:* Del. ex 13-15; Dupl. ex 13 *BRCA2:* Dupl. ex 4-11	Lim *et al.* (2007)
Spain	*BRCA2*	Yes	1.5%	-	Del. ex 2, ex 10-12, ex 15-16; Dupl. ex 20	Gutierrez-Enriquez *et al.* (2007)
USA	*BRCA1*	Yes	12.7%	-	Del. ex 14-20, ex 22, ex 13; Dupl. ex 13	Hendrickson *et al.* (2005)
USA	*BRCA1/2*	Yes	12%	-	*BRCA1:* Del. ex 1A/1B-2, ex 3, ex 8-9, ex 17, ex 20; Dupl. ex 13, among others	Walsh *et al.* (2006)
USA -Hispanic community	*BRCA1*	Yes	3.8%	-	Del. ex 9-12	Weitzel *et al.* (2007)

Prev *BRCA*: previously *BRCA*-negative patients by sequencing; Prevalence: prevalence of rearrangements in the families studied; Proportion: proportion of rearrangements in relation to all mutations. (*) In most of the studies, *BRCA* point mutations were not excluded. Del. = deletion, Dupl. = duplication, ex = exon, and Tripl. = triplication.
